# Stress relief treatment of aluminum/magnesium laminates fabricated by roll bonding technique

**DOI:** 10.1016/j.heliyon.2025.e41639

**Published:** 2025-01-02

**Authors:** Masoud Rashidi, Payam Tayebi, Ramin Hashemi

**Affiliations:** School of Mechanical Engineering, Iran University of Science and Technology, Tehran, Iran

**Keywords:** Al/Mg laminates, Annealing, Stress relief, Intermetallic, Recrystallization

## Abstract

Roll bonding of aluminum/magnesium laminates combines the good corrosion resistance of aluminum alloys with the beneficial mechanical properties of magnesium alloys. We studied the microstructure of aluminum Al-1051/AZ31 magnesium laminates fabricated by the roll-bonding process. The fabricated laminates were investigated in the as-fabricated condition and after subsequent stress relief annealing treatment at temperatures ranging from 200 °C to 400 °C. The investigation aims to determine the optimal temperature to decrease the residual stress of the severely deformed microstructure of the roll-bonded Al/Mg laminates. Internal residual stress can limit the application of such laminates. Such a thermal treatment enhances the interdiffusion of Al and Mg at the Al/Mg interface resulting in the formation of intermetallic phases. Intermetallic compounds are undesirable due to their brittleness and impact on the overall laminate structure and integrity. Detailed microstructure characterization of the intermetallic compounds and internal residual stress by means of recrystallization degree revealed that stress-relief treatment at 200 °C/1 h is sufficient to reduce internal residual stress and ensure adequate interdiffusion at Al/Mg interface to minimize the formation of brittle intermetallic compounds in the diffusion zone.

## 1 Introduction

Laminated metal composites combine exceptional mechanical properties with high corrosion resistance from different layers, enhancing material performance for critical applications [[1], [2], [3], [4]]. These composites benefit from various advantages of different metallic layers to address specific engineering challenges in sectors such as automotive and electronics [5]. Magnesium (Mg) alloys with low density and high specific strength and stiffness provide lightweight solutions for structural materials [6]. However, Mg alloys are vulnerable due to their susceptibility to corrosion, which restricts their broader application.

To overcome this limitation, laminates such as Al/Mg or Al/Mg/Al have been developed. Zhang et al. discussed the microstructure and mechanical properties of Al/Mg laminates where improvements in mechanical properties and electrical conductivity were achieved for accumulated roll-bonded Al and Mg composites [7]. Wang et al. employed a novel approach using corrugated rolling to achieve outstanding tensile properties in such Al/g laminates [8]. These laminates incorporate a corrosion-resistant aluminum (Al) layer, effectively utilizing the lightweight properties of Mg alloys while covering their drawbacks [[7], [8], [9], [10], [11], [12]]. Various manufacturing techniques, including rolling, casting, hot pressing, rolling extruding, and welding, have been explored to fabricate these composites [[7], [8], [9], [10], [11], [12], [13], [14]]. Among these, hot rolling is frequently preferred for Mg-containing laminates due to its cost-effectiveness and the specific benefits it offers in terms of activating the more challenging slip systems in Mg hexagonal close-packed (hcp) structures [12,15].

During the hot rolling process, the deformation of the hcp crystal structure in Mg alloys is facilitated by the increased mobility of dislocations. At higher temperatures, the primary slip system is activated and enables the material's deformation without fracturing [13,16]. Although hot rolling enhances ductility at elevated temperatures, it often introduces significant residual stresses that require subsequent annealing heat treatment to optimize the material for practical use [13,15]. Moreover, the non-uniform distribution of temperature and deformation during rolling may lead to heterogeneous microstructural features, including various dislocation densities and grain distortions. Hence, an annealing treatment is required to relieve the internal residual stresses and reduce the risk of immature mechanical failures accelerated by unwanted crack initiation and propagation [14,15]. On the other hand, as Hu et al. [16], and Y. Li [17] have demonstrated, annealing processes critically affect the microstructure and mechanical properties of laminates through diffusion at the interface between the Al and Mg. For instance, annealing at high temperatures may result in the formation of brittle intermetallic compounds at the Mg/Al interface, which can in turn impact the mechanical integrity of the laminates.

Despite advancements in the field, challenges remain in identifying the optimized process parameters and subsequent stress relief treatment to achieve the full potential of the Al/Mg laminates. This demands an in-depth analysis of the material microstructure to examine the formation of brittle intermetallic phases at the Al/Mg interface as well as the degree of recrystallization that indirectly indicates the residual stress in the laminated composites after heat treatment. As such, this study explores the microstructural developments in laminates composed of AZ31 Mg alloy and Al-1050 alloy, subjected to roll bonding and various annealing temperatures up to 400 °C to enhance our understanding of the Al/Mg laminates for critical applications. This investigation aims to study the *early-stage thermal treatment effects after the single-pass roll-bonding process to achieve an optimized balance between the heat treatment and structural integrity of such laminated composites.*

## 2 Experimental

In this research, laminates of Al-1050 and AZ31 were fabricated and subjected to a range of heat treatment conditions.

The nominal chemical compositions of these alloys are presented in Table 1.

**Table 1:** Nominal chemical composition of the two materials used in this study (in wt. %).

Material	Al	Si	Mg	Fe	Mn	Zn	Cu	Ti
AZ31	3.21	0.26	Bal.	–	0.2	1.12	0.08	–
Al1050	Bal.	0.1	0.05	0.22	–	0.02	0.03	0.01

In this investigation, AZ31 and Al-1050 sheets with thicknesses of 1 and 2 mm, respectively, were used to fabricate Al/Mg laminates. The roll bonding was performed on pre-heated sheets to 400 °C which minimizes cracking and increases the formability of the Mg sheets. The thickness reduction during the roll bonding process is approximately 50 %.

The roll bonding process results in accumulated residual stress in the laminates which limits their performance. Hence, a stress relief treatment is required to annihilate the residual stresses in the fabricated laminates and further enhance the metallurgical bonding between the layers. Such stress relief is done by annealing the laminates at typically 200–400 °C. Such a high-temperature annealing results in diffusion at the Al/Mg interface forming brittle intermetallic phases. In this investigation, a series of annealing treatments were performed at 200 °C, 250 °C, 300 °C, 350 °C, and 400 °C maintained for 1 h to find the optimum annealing temperature to achieve the best performance of the laminates. A reference sample was also included, which did not undergo any heat treatment after the roll bonding process. Table 2 lists the specimen IDs along with their respective treatment conditions.

**Table 2:** Specimen IDs and investigated annealing temperatures for stress relief treatment of fabricated AL/Mg laminates. The duration of annealing treatment is 1 h for all specimens.

Specimen ID	No-SR	SR-200	SR-250	SR-300	SR-350	SR-400
Annealing Temperature (°C)	–	200	250	300	350	400

Each sample was subjected to a series of characterizations to investigate the microstructural changes induced by the annealing processes. Cross-sections of the fabricated laminates were prepared following standardized sample preparation procedures, including mounting, grinding, and polishing to achieve a smooth and reflective surface suitable for metallographic investigation. Firstly, the prepared specimens were examined using optical microscopy (OM). Subsequently, the polished cross-sectional specimens were analyzed using an FEI Quanta 200 scanning electron microscope (SEM) equipped with a Schottky field emission gun (FEG). This instrument included an Oxford Inca energy dispersive X-ray detector (EDX) system for chemical composition analysis. Imaging was performed at acceleration voltages ranging from 5 to 20 kV. The SEM/EDX was utilized for local chemical analysis and elemental mapping of the interfacial areas on the cross-sections.

Selected specimens were further analyzed using Electron Backscatter Diffraction (EBSD) to gain detailed insights into the grain structure of the laminates. Settings for EBSD analysis included a step size range of 0.1–0.5 μm at an accelerating voltage of 20 keV. The analysis was performed using an EBSD system from Oxford Instruments, equipped with HKL Channel 5 software. Prior to EBSD analysis, the samples were polished using ion milling to ensure a high-quality, smooth surface, which is crucial for accurate EBSD measurements on Mg-alloy specimens. This preparation step allowed for optimal electron backscatter patterns and reliable data acquisition. The hardness of the interfacial transition zone of the Mg/Al composite plates was measured using a Vickers microhardness tester. The measurements were conducted with an applied load of 0.05 kgf for the best resolution at the Al/Mg interfacial zone.

## 3 Results

### 3.1 Optical microscopy of laminates

**Fig. 1:**
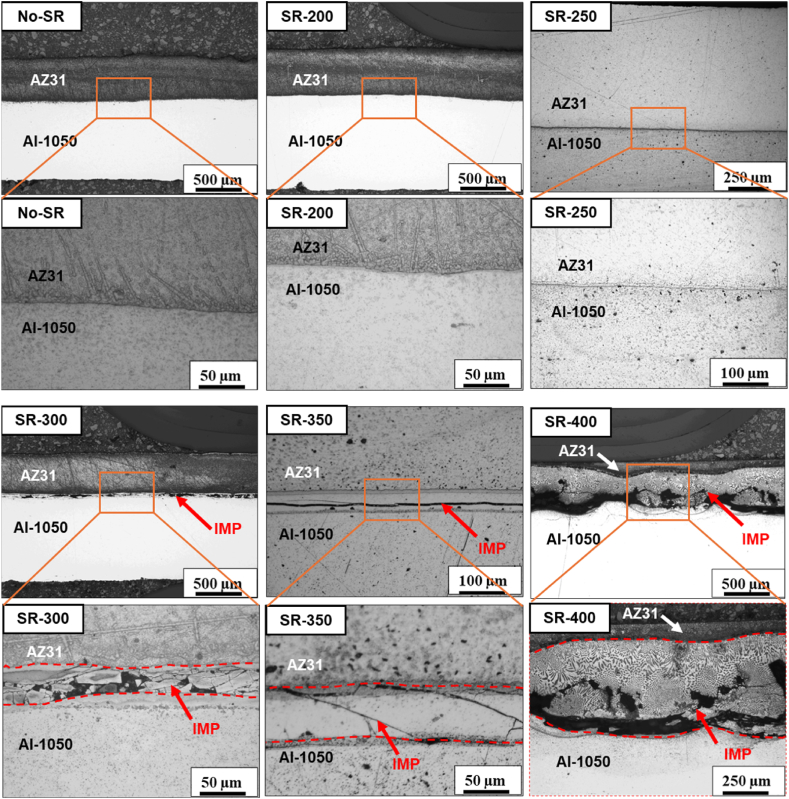
OM micrographs of the Al/Mg laminates for the No-SR, SR-200, SR-250, SR-300, SR-350, and SR-400 specimens at low and high magnifications showing the characteristics of the interface layer.

In the first step, an initial microstructural investigation of laminates was performed for specimens in the as-rolled condition (No-SR) and after annealing treatment at various temperatures according to Table 1. Fig. 1 shows OM micrographs highlighting the evolution of the Al/Mg interface at different annealing treatment temperatures. An overview and a zoomed-in view of the interface region are provided for each specimen. The micrographs show the development of brittle intermetallic phases (IMP; in Fig. 1 the area is marked with IMP and a red arrow) at different annealing temperatures.

For the No-SR specimen, the interface appears relatively clean with no contamination and oxides, confirming a proper preparation of the Al and Mg plates before the roll bonding procedure.Given that this specimen has not received any post-rolling thermal treatment, there is no expected diffusion at the interface, as confirmed by the OM images.

For the SR-200 and SR-250 specimens, low-temperature annealing, OM shows a very narrow <20 μm diffusion layer at the Al/Mg interface. For the SR-300, SR-350, and SR-400, a more pronounced diffusion layer is observed. The thickness of this diffusion layer increases by increasing the annealing temperature. The diffusion at the Al/Mg interface at such high-temperature treatments results in the formation of complex intermetallic phase compounds that are both brittle and hard. As can be seen, the IMP layer has fractured, and multiple cracks are visible in the OM micrographs. These cracks are the result of excessive pressure during the mounting of the specimen in the epoxy for the specimen preparation, grinding and polishing. Given that the specimens annealed at temperatures above 300 °C showed extensive formation of undesirable IMPs, as will be described further below, the following investigations were performed on specimens annealed at temperatures below 300 °C.

### 3.2 SEM-EDX of laminates

**Fig. 2:**
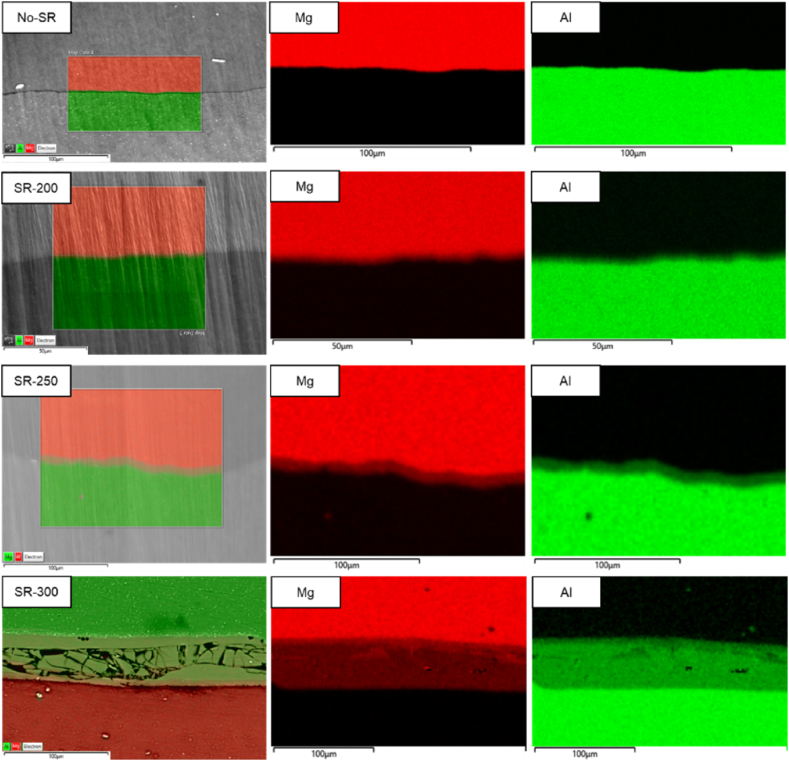
EDX Elemental Mapping of Al/Mg laminates for the No-SR, SR-200, SR-250, and SR-300. Only the EDX maps for the Mg and Al elements are shown.

**Fig. 3:**
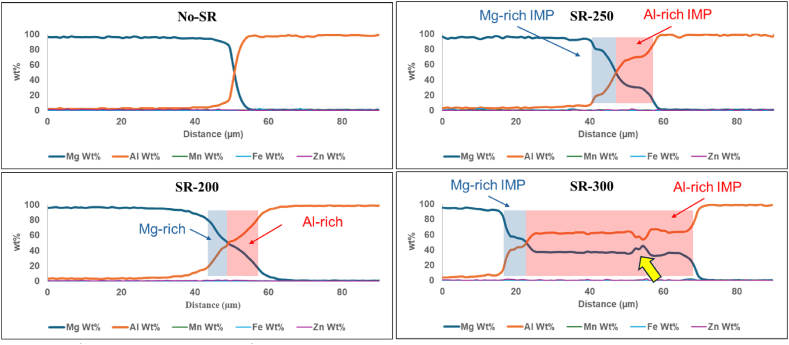
SEM/EDX line-scan analysis Al/Mg laminates for No-SR, SR-200, SR-250, and SR-300. The transparent blue and red boxes highlight the Mg-rich and Al-rich IMPs. The yellow arrow in the SR-300 line scan highlights an artifact caused by porosity, which occurred when a broken piece of the IMP layer fell out during sample preparation.

The SEM-EDX elemental mappings of the laminates are provided in Fig. 2. The EDX elemental mappings highlight the distribution of Al and Mg elements at the interfaces of the laminates. Moreover, EDX line scans are presented in Fig. 3 which provide insights into the quantitative distribution of the elements across the AL/Mg interface and the formation of various intermetallic compounds.

For the reference sample (No-SR), the SEM-EDX mapping shows a clear and sharp separation between the Mg and Al layers, which agrees well with the results presented in Fig. 1. The EDX maps show clear Mg and Al colorations with no signs of intermixing or diffusion, which aligns with expectations since the specimen hasn't undergone any additional heat treatment. In the EDX line scans, a narrow interface is visible for this specimen. It should be noted that in the EDX measurement, the interaction volume of the electron with the specimen is ≈ 2–3 μm for the settings used in this experiment (20 KeV), which in turn blurs the reported EDX composition at such a narrow interface between Al and Mg in the No-SR specimen. Hence, the variation in chemistry at this interface which covers an approximate 5 μm layer is due to the limitation of the technique and is not indicative of a diffusion between the Al-1050 and AZ31 at this stage.

After annealing treatment at 200 °C, in specimen SR-200, the EDX elemental maps indicate a slight interdiffusion of aluminum and magnesium layers ≈10 μm, the onset of diffusion processes. By increasing the annealing temperature to 250 °C, the thickness of the diffusion layer is slightly increased to ≈15 μm. In the EDX line scan, there are clear indications that the diffusion layer consists of two different chemistries of intermetallic phases, a relatively thinner Mg-rich IMP layer close to AZ31 and a thicker Al-rich IMP close to the Al-1050 layer, similar to the obtained results by Nie et al. on annealing treatment of Al/Mg laminates at 250 °C [12].

The sample treated at 300 °C, SR-300, shows more pronounced diffusion at the interface, with visible structural changes including cracks and voids due to the formation of brittle intermetallic compounds which cracked during sample preparation. The corresponding EDX map shows a significant intermixing of elements across approximately 75 μm which is an indication of a much faster kinetics of diffusion at such high temperatures. Similar to SR-250, in the EDX line scans, two different chemistries are characterized, a relatively thinner Mg-rich IMP layer close to AZ31 and a much wider Al-rich IMP layer close to the AL-1050. It is worth noting that the thickness of the Mg-rich IMP layer is not dramatically increased between the SR-250 and SR-300 conditions, while the thickness of the Al-rich IMP layer is significantly thicker in the SR-300 compared to SR-250. The area indicated by the yellow arrow in the SR-300 line scan is an artifact caused by porosity, which occurred when a broken piece of the IMP layer fell out during sample preparation.

### 3.3 EBSD analysis

EBSD analysis was performed on the No-SR, SR-200, and SR-250 specimens to further explore the effect of heat treatment on the grain structure, recrystallization degree, and the status of low-angle grain boundaries (LAGBs).

**Fig. 4:**
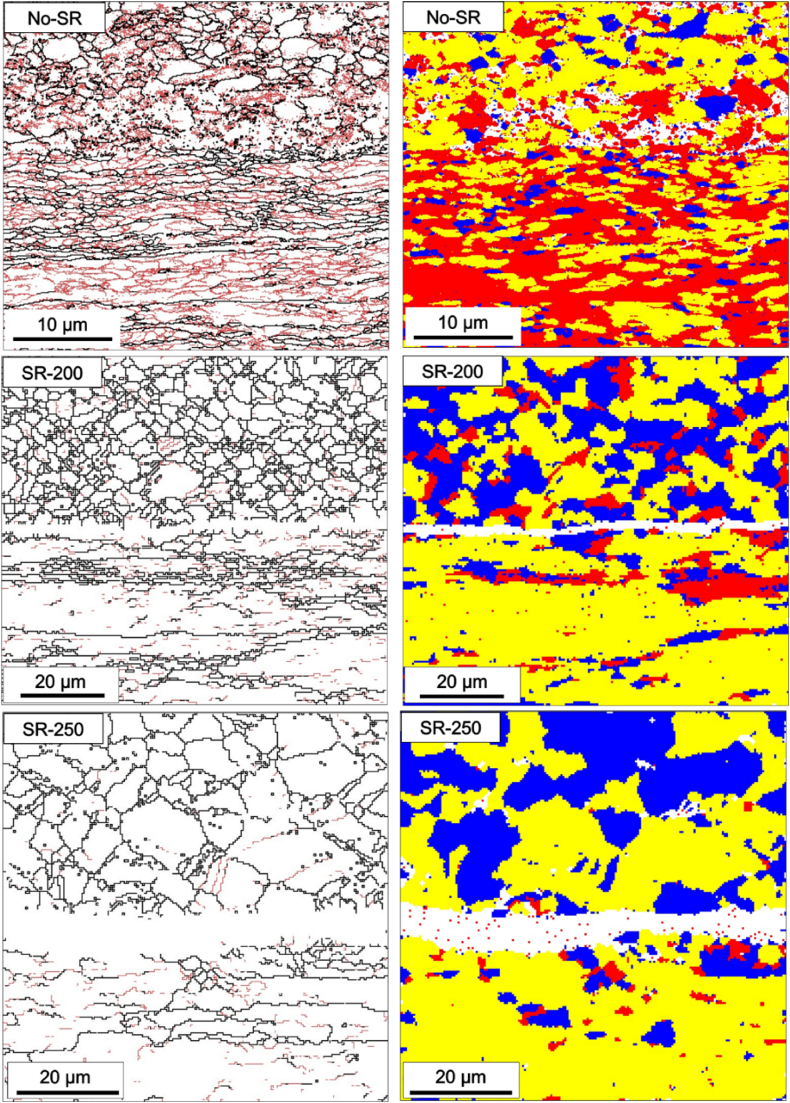
EBSD analysis of laminates showing grain structure for No-SR, SR-200, and SR-250 samples. In the left column pictures, grain boundary maps are presented where the thick lines indicate misorientation >10° (HAGBs), and red lines show the LAGBs with a misorientation of 2–10°. In the right column pictures, red, yellow, and blue colors show deformed, substructure, and recrystallized grains, respectively.

In Fig. 4, the obtained results from EBSD measurements are presented. On the left column pictures in Fig. 4, grain boundary maps are presented where high angle grain boundaries (HAGBs) (>10°), and LAGBs (2–10°) are shown using black lines, and red lines respectively. The HAGBs show the grain size and morphology while the LAGBs are representative of the plastic deformation because of roll bonding.

As can be seen, in the No-SR specimen, there is a high density of LAGBs (red lines) and rather elongated grains for both Al-1050 and AZ31 that stem from severe deformation during the roll bonding procedure. In the same specimen, the recrystallization map on the right column pictures reveals a rather high amount of plastically deformed grains with a red color parallel to the rolling direction. The absence of heat treatment after roll bonding in this specimen has preserved the deformation microstructure characterized by high dislocation densities and a high number of deformed grains.

EBSD analysis of SR-200 specimen is shown in the middle row of Fig. 4. The grain boundary map shows a significant decrease in the density of LAGBs (red lines). The newly formed recrystallized grains are shown by means of the development of fine equiaxed grain morphology as well as a larger fraction of recrystallized grains (blue color) for AZ31. These changes in the microstructure are an indication of the onset of a recovery process and an indirect sign of decreasing residual stresses in the specimens. It should be noted that thermal treatment at 200 °C has partially decreased the density of LAGBs in the Al-1050, however, has not resulted in a high fraction of recrystallization. This may be due to the fact that aluminum alloys require thermal treatment at much higher temperatures for recrystallization and the formation of new equiaxed grains.

For the SR-250 specimen (lower images in Fig. 4), grain boundary maps show slight changes in the density of LAGBs for both AZ31 and Al-1050 compared to SR-200. For AZ31, the grain size has slightly increased compared to the SR-200 specimen and the fraction of recrystallized grains has slightly increased with almost no severely deformed grain (red color) in the recrystallization map. For the Al-1050, few newly formed grains are visible (in blue color) which is an indication of the onset of recovery and recrystallization. The white regions in the recrystallization map (right column images of Fig. 4) represent areas that were not indexed, either due to poor Kikuchi patterns (a result of the heavily deformed regions in the No-SR specimen) or because the intermetallic phase at the interface was not indexed. The latter is particularly evident in SR-200 and SR-250 specimens at the Al/Mg interface, as we did not include the Kikuchi pattern of the intermetallic in the database for verification.

### 3.4 Hardness measurement

**Fig. 5:**
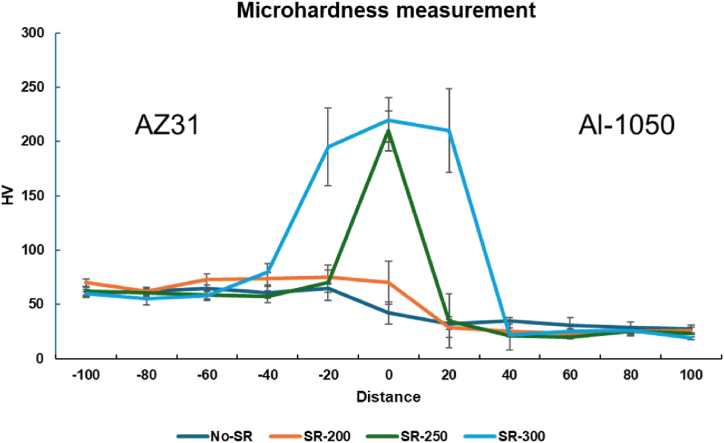
Microhardness profiles across AZ31/Al-1050 interface for No-SR, SR-200, SR-250, and SR-300 specimens summarizing the influence of annealing temperature on interfacial hardness.

The microhardness profile across the AZ31/Al-1050 interface is provided in Fig. 5. The hardness results are collected from at least three measurement profiles, and the average data with the standard deviation is presented in the graph.

The hardness measurements reveal distinct variations across the interface, reflecting the microstructural changes induced by the thermal treatments. The average values measured for the Al-1050 and AZ31 alloys are approximately 25 HV and 60 HV, respectively. For the No-SR specimen, there is no sudden increase in the hardness value at the interface which is in agreement with the reported microstructural investigations confirming the absence of IMP formation during the roll bonding process.

For the SR-200, the increase in the hardness is considerably lower than the hardness values obtained for SR-250, and SR-300. This may be due to a thin layer of the diffusion layer (almost equal size to the size of the hardness indent). Such a low hardness value may also indicate that no distinct IMP layer is yet formed and the interdiffusion between Al and Mg is not yet reaching the correct stoichiometry to form stable IMPs. For the SR-250 and SR-300 specimens, an increase in the hardness measurements is observed which confirms the formation of hard IMPs at such elevated temperatures.

For the measurements at the interface, a larger standard deviation is observed which is due to the brittle nature and local variation in the thickness of the intermetallic phase at the interface. Some contribution from the adjacent areas to the hardness of the interface layer is expected for the SR-200, SR-250, and SR-300. Overall, the microhardness measurements further confirmed the formation of significantly hard IMPs during the thermal treatment process after roll bonding.

## 4 Discussion

In our previous studies [18,19], we investigated the formability of composite bi-metallic laminates using both experimental and numerical methods. Subsequently, we identified the necessity to explore optimized process parameters during the preparation stage of the laminates before their final production into various geometries. The optimum goal of this investigation is to get insights on how to best prepare such laminates for better formability to a final geometry. Severe deformation during roll bonding induces a high amount of residual stress which limits its application. To decrease the residual stress of the laminates, an annealing treatment is required. Such a thermal treatment also enhances the metallurgical bonding (through interdiffusion of Al and Mg) between the laminated structures. During such annealing treatment, several metallurgical phenomena occur that are greatly affected by the treatment temperature, see e.g., Refs. [17,[20], [21], [22], [23], [24]]. Hence, this paper investigated laminated structures in the as-rolled condition and after thermal treatments at intervals of 50 °C, ranging from 200 °C to 400 °C, aiming to explore the optimized parameters that could result in formable Mg-Al laminates.

Interdiffusion and formation of the intermetallic phases at the Al/Mg interface were investigated by employing microscopy and microhardness analysis across the interfacial region of the laminates produced by various annealing parameters. Our finding indicates that there is no IMP formation in the as-rolled condition. After annealing at 200 °C, interdiffusion at the Al/Mg interface occurs, however, microhardness and EDX line scans suggest that the product of such diffusion is mainly in the form of a solid solution rather than a hard “layer” with a chemistry that matches or is close to the stoichiometry of Al_3_Mg_2_ or Mg_17_Al_12_. Our results do not exclude the possibility of forming IMP at a sub-micron-sized layer or in the form of IMP particles considering the limitation of the EDX resolution governed by the electron-specimen interaction volume. According to the Al-Mg binary alloy phase diagram [25], IMPs are thermodynamically stable at temperatures as low as 200 °C. However, the limited or unclear indication of these IMPs at 200 °C in our experimental conditions may be attributed to kinetic factors. At this annealing condition of 200 °C/1 h, the diffusion rates of Al and Mg atoms may be insufficient to overcome the activation energy barrier required for the extensive nucleation and growth of intermetallic compounds. Thus, despite the thermodynamic possibility of their formation, kinetic constraints prevent the development of IMP layers in the annealing time frame, causing both Al and Mg to remain in their respective solid solution phases (α-Al and α-Mg).

After annealing at 250 °C, quantitative EDX results and microhardness measurement confirm the presence of hard Al-rich IMPs (possibly Al_3_Mg_2_) close to Al-1050, and Mg-rich IMPs (possibly Mg_17_Al_12_) adjacent to the AZ31 plate. Increasing the annealing temperature to 300 °C significantly increases the kinetics of diffusion resulting in a much wider IMP zone. Our EDX analysis shows a thicker layer of Al_3_Mg_2_ on the Al side and a relatively thinner layer of Mg_17_ Al_12_ on the Mg side. The rapid growth rate of Al_3_Mg_2_ over Mg_17_Al_12_ may be attributed to a faster diffusion rate of Mg in Al than that of Al in Mg [26]. Moreover, the activation energy for the formation of Al_3_Mg_2_ has been suggested to be lower than that for Mg_17_Al_12_ [27].

The EBSD analysis utilizing grain boundary and recrystallization maps revealed valuable insights into the development of the grain structure during different annealing temperatures. In the as-rolled condition, the microstructure consists of heavily deformed and elongated grains parallel to the roll bonding direction. After annealing treatment at 200 °C, the microstructure developed into a more equiaxed grain structure with much lower LAGBs. It should be noted that the recrystallization of the Mg-alloys occurs at approximately 200°C–250 °C depending on the various parameters such as the degree of stored energy from the previous plastic deformation. Comparing the EBSD data for SR-200 and SR-250 shows that for AZ31, increasing the annealing temperature to 250 °C does not necessarily result in a higher degree of recrystallization and/or dislocation annihilation (to reduce internal residual stress). Moreover, annealing at 250 °C results in a slight grain growth in the AZ31. For the Al-alloys recrystallization occurs at much higher temperatures of approximately 350–400 °C. As can be seen in the EBSD results in Fig. 4, the density of LAGBs has significantly decreased during the annealing treatment, however, no major recrystallization has occurred for the Al-1050 and not many newly formed equiaxed grains are visible.

Considering that Al-1050 in the laminate composite is mainly responsible for the corrosion resistance and most of the structural properties are related to the Mg AZ31 alloy, the annealing treatment should be designed to optimize the grain structure for the AZ31 alloy. Hence, it is believed that the annealing treatment at 200 °C for 1 h can provide both sufficient bonding between the laminated structure through interdiffusion at the Al/Mg layer, and a sufficient degree of recrystallization to improve the ductility and strength of AZ-31 alloy. Moreover, annealing at 200 °C prevents excessive formation of hard and brittle intermetallic compounds.

## 5 Summary and conclusion

In this paper, we investigate the roll bonding of pre-heated AZ31 and Al-1050 sheets to 400 °C for the fabrication of crack-free laminated structures. The results indicate that for the optimization of the annealing temperature in the roll bonding process of Al-1050 and Mg AZ31, the primary focus should be on minimizing the formation of intermetallic compounds (IMCs) due to their hard and brittle characteristics. Our findings indicate that at 200 °C, no significant IMC formation occurs, making this temperature favorable for avoiding brittle phases while providing effective stress relief and metallurgical bonding thanks to the interdiffusion of Al and Mg. At annealing temperatures of 250 °C and above, distinct hard and brittle IMPs of Al_3_Mg_2_ and Mg_17_Al_12_ form on the Al and Mg sides, respectively. Moreover, such a high annealing temperature accelerates grain growth for the AZ31 with no significant addition of recrystallization. Such grain growth can substantially degrade mechanical properties. Therefore, the optimal annealing temperature range for achieving a balance between effective stress relief and minimal IMC formation is 200 °C. This ensures the structural integrity and performance of the Al-1050/Mg AZ31 laminate for practical applications.

## CRediT authorship contribution statement

**Masoud Rashidi:** Writing – original draft, Visualization, Validation, Software, Methodology, Investigation. **Payam Tayebi:** Writing – review & editing, Validation, Supervision, Project administration, Conceptualization. **Ramin Hashemi:** Writing – review & editing, Validation, Supervision, Methodology, Investigation, Data curation, Conceptualization.

## Declaration of competing interest

The authors declare that they have no known competing financial interests or personal relationships that could have appeared to influence the work reported in this paper.
